# Exploration of Body Self-Image and Associated Body Composition Supplement Behaviors in College Students

**DOI:** 10.3390/nu18010007

**Published:** 2025-12-19

**Authors:** Jennifer L. Berridge, Aleah Austin, Shannon N. Clifford, Sarah P. Shultz

**Affiliations:** 1 Department of Health and Human Performance, Fort Lewis College, Durango, CO 81301, USA; berridgejennifer8@gmail.com; 2 Department of Public Health, Fort Lewis College, Durango, CO 81301, USA; 3 Marjorie K. Unterberg School of Nursing and Health Studies, Monmouth University, West Long Branch, NJ 07764, USA; scliffor@monmouth.edu

**Keywords:** health behavior, psychological factors, dietary supplements, body image

## Abstract

**Background/Objectives**: Body self-image interacts with young adults’ health behaviors in complex ways, yet its role in shaping supplement use is not well defined. This study examined young adults’ body self-image and their use of dietary supplements commonly marketed to enhance physique, fitness, and weight outcomes. **Methods**: College students (N = 122; 18–28 years) completed the Body Self-Image Questionnaire-Short Form to assess body self-image and answered three dichotomous questions related to general dietary (DS), performance-enhancing (PES), and weight-loss (WLS) supplement use. Student scores were calculated across nine psychological subscale groups and ranked as high, moderate, or low percentile within our study sample. Chi-square analyses examined supplement prevalence in student responses ranked within the highest and lowest tertiles; middle tertile data were excluded as part of the extreme-group design. All responses were included in the logistical regression analysis. **Results**: Higher scores for the Investment in Ideals and Health-Fitness Evaluation subscales observed significantly higher PES use; both subscales and the male gender were significant predictors of PES use. Higher scores for the Fatness Evaluation, Negative Affect, and Social Dependence subscales observed significantly higher WLS use; Fatness Evaluation and age were significant predictors of WLS use. Subscales were not significantly associated with general supplement use in chi-square or logistical regression analyses. **Conclusions**: Our findings suggest that internalized appearance ideals, emotional distress, and social approval pressures are linked to weight- and physique-driven supplement behaviors in college students. Targeted, inclusive campus wellness initiatives are needed to address psychological drivers of weight-management practices, including potentially high-risk supplement use.

## 1. Introduction

Body self-image in young adults remains a central focus of public health and behavioral research due to its strong associations with self-esteem, mental health, and health-behavior adoption. These links between body self-image, emotional well-being, and health-related behaviors have been repeatedly demonstrated in college populations [1,2].

College life intensifies appearance pressures, which can be shaped by cultural expectations and social media imagery that reinforce perfectionistic ideals [3]. Research has revealed important gender-specific and psychosocial differences in how these ideals are internalized. Ghaderi et al. [4] identified that the drive for muscularity and body dissatisfaction strongly predict supplement use among young men, while Kelly et al. [5] found that self-compassion buffers body image distress and disordered eating among women. Persistent body dissatisfaction and social comparison, exacerbated by digital media exposure, can heighten anxiety and depressive effects and foster reliance on unhealthy coping mechanisms such as extreme dieting, over-exercise, or supplement misuse [6,7]. Understanding how specific psychological dimensions of body self-image influence supplement-related behaviors is essential for developing targeted interventions that address the motivational drivers underlying these practices.

College students frequently pursue weight-management and body composition goals under strong sociocultural pressures to attain an “ideal” physique [3]. Lower body image satisfaction is consistently linked to weight-control behaviors such as restrictive dieting, compulsive exercise, and dietary supplement use [8]. Appearance- and performance-enhancing substance use has been shown to occur concurrently with disordered eating symptomatology among U.S. college students, underscoring the broader psychosocial and health risks of physique- and weight-driven practices [9]. When body insecurity intersects with low self-efficacy, students often turn to supplement-based strategies to manage their weight or modify their body composition to align with internalized appearance ideals and peer norms [4,10,11]. The commercial dietary supplement market has expanded to simultaneously meet and exploit these vulnerabilities, promoting products that emphasize control, enhancement, and self-optimization [12,13]. Large-scale survey data indicate that approximately two-thirds of U.S. college students use dietary supplements at least weekly, with many consuming multiple supplement categories concurrently [14].

While many students report general dietary supplement (DS) use for health maintenance [12], performance-enhancing supplements (PES) and weight-loss supplements (WLS) pose greater concerns for weight-management and body composition outcomes. These products often feature unverified claims and undisclosed ingredients and may introduce toxicities or contamination risks [15,16,17]. The NIH Office of Dietary Supplements [18] further warns that many PES and WLS agents lack clinical efficacy and can interact adversely with medications or exacerbate existing conditions. Moreover, Nagata et al. [19] observed that early use of protein powders and muscle-building supplements during adolescence predicts progression to more hazardous performance-enhancing drugs, indicating a behavioral continuum that begins with normalizing the use of supplements. Despite growing recognition of the risks associated with these supplements and their potential for behavioral escalation, limited research has examined which body self-image constructs specifically predict different categories of supplement use among college students.

The intersection between unregulated, over-the-counter supplement access, psychological distress, and low body image satisfaction [1] highlights an urgent need for evidence-based, holistic interventions on college campuses. Social media environments and peer norms continue to amplify appearance-based comparisons and physique anxiety across gender identities [7]. Taking a novel approach to bridge the gap in research, this study explored whether specific Body Self-Image Questionnaire-Short Form (BSIQ-SF) subscales are associated with the self-reported use of DS, PES, and WLSs among college students. We hypothesized that subscales reflecting internalized ideals would predict PES use, while subscales capturing affective distress would predict WLS use. We believe that these findings could offer insight into the psychological mechanisms driving supplement-based weight-management behaviors and inform mental-health interventions within collegiate wellness settings.

## 2. Materials and Methods

### 2.1. Study Design

This cross-sectional study explored differences in categorized dietary supplement use across varying levels of body self-image subscales among college students at a Native American-Serving Non-Tribal Institution. Given limited prior research examining the associations between specific body self-image constructs and categorized supplement use, this study was designed to identify preliminary associations that can inform future research. The study was approved for exemption status by the Institutional Review Board of Fort Lewis College (Durango, CO, USA). Participants provided implied digital consent prior to survey initiation.

Participants and recruitment-eligible participants were 18–28 years old and enrolled in at least one college course at a Native American-Serving Non-Tribal Institution within rural Colorado. Recruitment efforts included campus flyers, digital and classroom announcements, social media, and outreach through campus health and wellness organizations to promote a diverse cohort of survey responses. Participants were not incentivized to complete the survey.

### 2.2. Data Collection

Participants accessed the encrypted, de-identified online survey via a QR code (22 January–14 March 2025) (File S1). Respondents self-reported demographic data (i.e., age, race, and gender identity). Body self-image was assessed via the validated Body Self-Image Questionnaire-Short Form (BSIQ-SF) [20,21,22,23]. The BSIQ-SF was cross-validated against the longer 51-item version (α = 0.68–0.92) [23] in large college samples and demonstrated an acceptable model fit (CFI = 0.93; NNFI = 0.92; RMSEA = 0.04) [20]. BSIQ-SF responses were scored according to published author instructions, and subscores were calculated for the following: Overall Appearance Evaluation (OAE), Health Fitness Influence (HFI), Investment in Ideals (II), Health-Fitness Evaluation (HFE), Attention to Grooming (AG), Height Dissatisfaction (HD), Fatness Evaluation (FE), Negative Affect (NA), and Social Dependence (SD). Additionally, respondents answered three dichotomous questions related to the ingestion of legal over-the-counter ergogenic dietary supplements during the previous 12 months; these questions were intentionally selected to identify the psychological predictors of supplement behavior rather than to characterize clinical consumption patterns. Participants confirmed use of general dietary supplements (DS) [16], performance-enhancing supplements (PES) [17], or weight-loss supplements (WLS) [18] within the last 12 months. Each question provided participants with specific product examples to improve classification accuracy. DS were defined as vitamin or mineral supplements, with examples including multivitamins and individual vitamins (B-Complex, B12, B6, C, A, D, E, and K) and minerals (calcium, magnesium, potassium, iron, and zinc). WLS were defined as weight-loss supplements, with examples including appetite suppressants, thermogenic agents (caffeine pills and green tea extract), and fat blockers. PES were defined as performance-enhancing supplements, with examples including creatine, beta-alanine, branched-chain amino acids (BCAAs), and caffeine pills. These definitions and product categories were drawn from the U.S. Food and Drug Administration (FDA) classification criteria and data from the National Health and Nutrition Examination Survey (NHANES) [12]. A 12-month recall period for supplement use was selected to balance participant memory accuracy with the need to capture behaviors that may be intermittent or cyclical in nature. This approach aligns with best practices for self-report surveys, as longer intervals can improve internal validity when the behavior is irregular and meaningful over time [24]. A ‘yes’ response was scored as 1 and a ‘no’ response was scored as 0 for each item. Numeric responses were analyzed both descriptively and through inferential models, which included all continuous BSIQ-SF subscale scores.

### 2.3. Data Processing and Analysis

Incomplete or ineligible survey responses were excluded if any required item was unanswered. Surveys with missing data were excluded to support complete-case modeling in regression analyses and to ensure the accurate classification of supplement use. This protocol followed accepted practices for complete-case analysis when data are assumed to be missing completely at random [25]. Chi-square testing using an extreme-groups design was conducted as an exploratory method to identify large contrasts in supplement use across psychological profiles [26]. For exploratory heat-map visualization and contingency table analyses, BSIQ-SF subscale scores were stratified into low (<40th percentile) and high (>60th percentile) categories. Participants in the middle range were only excluded from chi-square testing and were retained in all multivariable regression analyses. This design was used solely to visualize high-risk clustering patterns and was not considered the primary analytic strategy. Theoretical results indicate that the top–bottom third contrast retains ~80–90% of the efficiency for trend detection [26]. Chi-square analysis examined the prevalence of supplement use (DS, PES, and WLS) in participants within high- and low-scoring groups for descriptive comparisons. Fisher’s exact test was applied when expected cell counts were small. Post hoc analysis of adjusted residuals exceeding ±2.0 indicated that the observed frequencies deviated meaningfully from the expected values. Because dichotomization reduces statistical efficiency and can distort effect estimation, all hypothesis testing was conducted using continuous scores in logistic regression models. Multivariable logistic regression served as the primary inferential method for all outcomes. Supplement use served as the dependent variable, continuous BSIQ-SF subscores and age were modeled as predictor variables, and race/gender identities were included as categorical covariates where appropriate. Model construction was theory-driven to preserve appropriate events-per-variable ratios, particularly for WLS outcomes where prevalence was low, consistent with recommended practices that prioritize substantive knowledge over automated variable selection to reduce overfitting and improve model stability [27]. Multicollinearity was evaluated using variance inflation factors (VIFs), and no predictors exceeded conventional thresholds of concern. Sensitivity analyses were conducted using alternative predictors, when warranted, to evaluate model stability. Descriptive statistics and chi-square analyses were performed in IBM SPSS Statistics version 28 (IBM Corp., Armonk, NY, USA); all regression analyses were completed with JASP (University of Amsterdam, Amsterdam, The Netherlands). Odds ratios (ORs), 95% confidence intervals (CIs), and pseudo R^2^ values were extracted for interpretation. Statistical significance was set at *p* < 0.05 for all analyses.

## 3. Results

Survey responses were collected from 125 participants. Incomplete responses (N = 3) were excluded; valid data were available for 122 participants (97.6%). Participants primarily identified as female (65.5%; N = 80), while 29.5% identified as male (N = 36), and 5% identified as non-binary (N = 6). Age distribution ranged from 18 to 28 years. The most commonly reported ages were 20 (18.9%; N = 23) and 21 (18.9%; N = 23). The majority of participants identified as White or Caucasian (53.3%, N = 65) and American Indian or Alaska Native (34.4%, N = 42). Hispanic or Latinx (8.2%, N = 10), Asian (1.6%, N = 2), Black or African American (1.6%, N = 2), and Native Hawaiian or Other Pacific Islander (0.8%, N = 1) were also represented. Varying percentages of participants reported using DS (69%), PES (60%), and WLS (17%).

**Figure 1:**
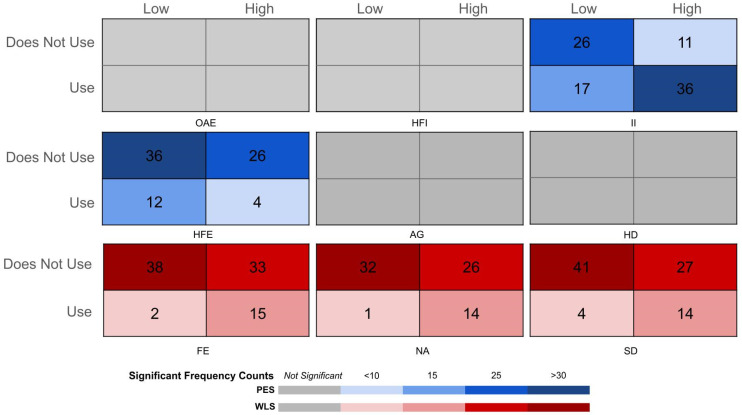
Heat map visualizing supplement use distribution across psychological subscale categories. Heat maps illustrate the distribution of dietary supplement use, including dietary supplements (DS), performance-enhancing substances (PES), and weight-loss supplements (WLS) across participant groups categorized by psychological subscale scores (low/high). Subscale categories are as follows: OAE = Overall Appearance Evaluation; HFI = Health Fitness Influence; II = Investment in Ideals; HFE = Health-Fitness Evaluation; AG = Attention to Grooming; HD = Height Dissatisfaction; FE = Fatness Evaluation; NA = Negative Affect; and SD = Social Dependence. Red shading indicates statistically significant observations of weight-loss supplement (WLS) use; blue shading indicates statistically significant observations of performance-enhancing substance (PES) use. The intensity of shading indicates cell frequency so that lighter/darker shades are associated with lower/higher cell counts within that contingency table, respectively. The legend displays the numerical ranges corresponding to each gradient color when findings reach statistical significance, with gray indicating non-significant findings for any category of supplement use. These heat maps are intended to visualize clustering patterns only; inferential testing was conducted using multivariable regression models with continuous predictors.

Neither chi-square testing nor multivariable regression revealed significant predictors of general dietary supplement use. Exploratory chi-square analyses showed a higher prevalence of PES use among students scoring high on the II (χ^2^ (1) = 12.740, *p* < 0.001) and HFE (χ^2^ (1) = 8.135, *p* = 0.005) subscales (Table 1). While both high and low groups included individuals who did not use supplements, adjusted residuals indicated that a noticeably greater proportion of PES users came from the high-scoring groups within the II (76.6%) and HFE (76.7%) subscales (Figure 1). Both Investment in Ideals and Health-Fitness Evaluation independently predicted PES use within the multivariable logistic regression using continuous subscale scores (Table 2). Each one-point increase in II corresponded to a 34% increase in PES odds (OR = 1.34, 95% CI [1.14–1.58], *p* < 0.001), while HFE was associated with a 28% increase per point (OR = 1.28, 95% CI [1.11–1.48], *p* = 0.001). Male gender was a strong independent predictor (OR = 3.40, 95% CI [1.53–7.54], *p* = 0.003). No other subscales independently predicted PES use. Age was not significant.

Chi-square analysis indicated higher WLS prevalence among students scoring high on the FE (χ^2^ (1) = 9.646, *p* = 0.002), NA (χ^2^ (1) = 11.320, *p* < 0.001), and SD (χ^2^ (1) = 8.270, *p* = 0.007) subscales (Table 1). Adjusted residuals indicated that a strong majority of participants who scored high in the FE subscale (95%), NA subscale (97.0%), and SD subscale (91.1%) also reported WLS use (Figure 1). In regression modeling, only FE remained a robust predictor of WLS use (OR = 1.36, 95% CI [1.16–1.59], *p* < 0.001) (Table 2). Each additional year of age increased WLS odds by 23% (OR = 1.23, 95% CI [1.00–1.51], *p* = 0.046). NA and SD did not retain significance after adjustment in multivariable models. All other subscales did not independently predict WLS use.

Model fit was strong for both PES (pseudo R^2^ = 0.19) and WLS (pseudo R^2^ = 0.22) regressions, indicating moderate explanatory value.

## 4. Discussion

Distinct domains of body self-image were associated with specific supplement behaviors, illustrating how psychological factors map into nutrition-related decision making in college populations. As hypothesized, both exploratory group contrasts and multivariable regression analyses converged on Investment in Ideals (II) and Health-Fitness Evaluation (HFE) as central predictors of PES use. Regression modeling demonstrated that each one-point increase in II and HFE was associated with approximately 30–35% higher odds of PES use, independent of other covariates, highlighting the salience of ideal internalization and self-evaluation in physique-driven supplement behaviors. Prior studies similarly report that individuals with heightened concern for idealized physiques are more likely to engage in PES behaviors [3,17]. Elevated II and HFE scores may capture a motivation profile centered on optimization, where physique enhancement becomes a proxy for competence, confidence, or identity. These findings align with broader patterns observed in fitness-oriented populations, where high supplement prevalence and heterogeneous product combinations are common [28]. Mazzilli et al. reported that 85% of gym users consumed dietary supplements, with substantial variability in types and combinations, often through self-directed rather than professional guidance. The 60% PES prevalence in our college sample suggests that appearance- and performance-driven supplementation represents a normative weight-management strategy among students with elevated body image investment, potentially influenced by fitness culture and peer norms. Media-driven norms and peer reinforcement can further intensify these tendencies, equating visible muscularity or leanness with self-worth [10]. In practice, II and HFE patterns can serve as quantifiable and clinically relevant indicators for nutrition or health providers seeking to identify appearance-driven supplement use as an influencing factor for weight management.

Conversely, extreme-group comparisons suggested elevated WLS prevalence among students high in FE, NA, and SD; however, when modeled concurrently, FE emerged as the dominant psychological predictor of WLS use, with age contributing additional risk. These subscales reflect body fat dissatisfaction, emotional distress, and reliance on social approval, which are psychological states that can promote maladaptive coping behaviors [6,11]. These findings indicate that dissatisfaction with perceived body fatness is more proximally related to WLS behavior than emotional distress or social dependence alone once overlapping variance is considered. Similar patterns have been described in prior research linking body dysmorphic tendencies to emotional distress and substance misuse [11], as well as reports of the toxicity, contamination, and physiological risk associated with weight-loss agents [15]. The cultural environment, saturated with messages of thinness and self-optimization, amplifies these pressures and normalizes extreme methods of control [6]. Within this context, affective and social influences converge on behaviors that prioritize external validation over sustainable health pursuits and practices associated with weight management. General DS use, by contrast, did not remarkably correlate with body self-image subscales, suggesting that motivations for DS consumption are likely health-oriented rather than appearance-driven [12]. This distinction is critical for both counseling and education, as it highlights the need to differentiate between evidence-based supplementation for nutritional adequacy or general health and wellness behaviors and maladaptive use linked to weight or physique management and control. Educational strategies that normalize clinically indicated supplement use while addressing the risks and misinformation surrounding PES and WLS can reduce stigma and promote informed, health-centered behaviors [17]. Importantly, PES and WLS can displace evidence-based dietary strategies, emphasizing the need for nutritional interventions to reinforce food-first approaches that focus on micronutrient adequacy, protein distribution, training-aligned fueling, and recovery-supportive eating patterns for weight management. Clear communication is essential, as over-the-counter agents typically lack demonstrated efficacy, may introduce contamination or toxicity risks, and can divert students from the foundational nutrition behaviors necessary for safe and effective body composition management [15,16,17,18].

This study was exploratory in nature and is not without limitations. A priori sample size was calculated and met within the data collection window. Although the overall sample size (N = 122) supported the primary regression analyses, the use of extreme-group stratification was restricted to exploratory chi-square visualization and reduced the number of observations in some contingency table cells. Therefore, inference was based on regression modeling using continuous subscale scores, which retained all available data. Fisher’s exact test was applied where expected cell counts were small, and hypothesis testing relied on regression models with adequate events-per-variable ratios, particularly for PES outcomes.

While extreme-group analyses aided the interpretability and visualization of risk clusters, regression models were prioritized to preserve statistical efficiency and minimize bias due to dichotomization. Anthropometric measures (e.g., height, weight, and BMI) were deliberately excluded from the survey due in part to their limitations in psychological and behavioral research. Research shows that BMI feedback can increase body dissatisfaction and unhealthy behaviors, especially among college students [29,30]. Given that BMI-related metrics are strongly associated with societal health standards [8], their exclusion may have encouraged participants, especially those with body image concerns, to provide more accurate responses uninfluenced by normative physical benchmarks. Also, while participants were provided with specific product examples for each supplement category, the potential for misclassification bias remains. Some products contain multiple ingredients spanning categories (e.g., pre-workout supplements with caffeine and BCAAs), and certain items were listed in multiple categories to reflect dual marketing purposes (e.g., caffeine pills in both the WLS and PES categories). This overlap may have introduced ambiguity in reporting. The dichotomous yes/no format captured the presence or absence of use but did not assess dosage, frequency, or specific product brands, limiting the interpretation of use patterns. Despite these constraints, the provision of concrete examples likely improved reporting accuracy compared to categorical labels alone. The 12-month recall period for supplement use may have introduced recall bias, particularly if use was infrequent or occurred early in the reporting window. Students may have underreported or misremembered sporadic supplement use, potentially attenuating the observed associations. However, this extended timeframe was selected to capture intermittent or seasonal patterns of use that shorter recall periods might miss [24], and the prevalence rates observed align with those reported in other large-scale college supplement surveys [14]. The relatively low prevalence of WLS (17%) necessarily constrained the model’s complexity for that outcome; therefore, WLS regression models were purposefully parsimonious. Although precision for WLS estimates was more limited than for PES, the observed effects were directionally consistent and clinically interpretable.

Sample demographics were largely consistent with Fort Lewis College’s diverse student body, particularly for American Indian/Alaska Native (AI/AN) representation (34.4% in sample vs. 24% campus-wide), reflecting the institution’s designation as a Native American-Serving Non-Tribal Institution [31]. Importantly, the inclusion of non-binary participants and AI/AN students are notable strengths, as these populations are historically underrepresented in body image and dietary supplement research [32]. However, some variation within other race classifications was observed. Hispanic/Latino students were underrepresented (8.2% vs. 14.4% campus), while White students were slightly overrepresented (53.3% vs. 44.1% campus). Additionally, the percentage of the Black students surveyed aligns with representation on campus (1.6% vs. 2% campus); however, this percentage is notably low (1.6% vs. 12.1% nationally) and cannot accurately reflect this community’s dietary supplement needs [31]. While race was not a predictor of supplement use, the strong prevalence of AI/AN students (34.4% of sample) underscores the need for culturally grounded campus nutrition interventions that integrate cultural knowledge systems while addressing unique health disparities experienced by historically underrepresented populations [31,32].

Despite these limitations, this study offers methodologically sound preliminary evidence regarding psychological contributors to physique- and weight-driven supplement behaviors in college students.

## 5. Conclusions

In this diverse college sample, distinct psychological dimensions of body self-image were linked to specific categories of supplement behavior, clarifying how internalized ideals, affective distress, and social approval pressures influence weight-management practices. Higher Investment in Ideals and Health-Fitness Evaluation scores aligned with physique-oriented PES use, whereas elevated Fatness Evaluation scores independently predicted WLS use. Conversely, general DS use appeared driven by health maintenance rather than physique- or weight-driven motives. These patterns underscore how body self-image constructs shape students’ decisions to adopt appearance- or weight-focused supplement strategies and highlight the importance of identifying at-risk groups earlier through psychosocial screening and nutrition assessments. The presence of weight- or physique-driven motives should prompt practitioners to redirect students toward food-first, periodized nutrition, and realistic expectations for body composition changes [1,17]. Integrated approaches that promote mental well-being, self-acceptance, and safe supplement practices [2,3], along with self-compassion strategies that buffer body dissatisfaction [5], may be particularly meaningful in reducing the reliance on high-risk products. At the systems level, strengthening supplement-verification standards, teaching label and marketing literacy, and training campus personnel to recognize supplement-related risks [15,16,17,18], particularly in environments that heighten appearance comparisons [6,7], may further reduce harmful weight-management behaviors. Collectively, these findings underscore the need for coordinated, evidence-based, and culturally responsive approaches that advance a comprehensive, nutrition-centered integrated health care model of weight management. Future campus models should endeavor to prioritize mental well-being, inclusivity and programs led by trained providers that proactively educate and offer prevention, health promotion, and intervention strategies aimed at reducing or eliminating the reliance on high-risk supplement use across college populations.

## Figures and Tables

**Table 1:** Exploratory prevalence of supplement use across psychological extreme groups. Extreme-group stratification (low < 40th percentile; high > 60th percentile) is presented to visualize clustering patterns in supplement use across the BSIQ-SF subscales. Inferential testing was conducted using multivariable logistic regression models with continuous predictors. Subscale categories are as follows: OAE = Overall Appearance Evaluation; HFI = Health Fitness Influence; II = Investment in Ideals; HFE = Health-Fitness Evaluation; AG = Attention to Grooming; HD = Height Dissatisfaction; FE = Fatness Evaluation; NA = Negative Affect; and SD = Social Dependence. *p*-values have been calculated using Fisher’s exact test; bold font indicates statistically significant differences (*p* < 0.05). Upper and lower limits of expected distribution are found in italicized font.

	High/Use	Low/Use	High/Not Use	Low/Not Use	*p*-Value	Min Count
Dietary Supplement						
OAE	27	25	8	15	0.142	10
	*(21–29)*	*(23–31)*	*(7–15)*	*(8–23)*		
HFI	28	15	11	10	0.416	8
	*(23–30)*	*(12–20)*	*(9–16)*	*(5–12)*		
II	35	28	12	15	0.365	12
	*(29–37)*	*(26–34)*	*(10–18)*	*(9–17)*		
HFE	21	31	9	17	0.805	10
	*(16–24)*	*(28–36)*	*(6–14)*	*(12–20)*		
AG	31	28	8	15	0.218	10
	*(24–32)*	*(27–35)*	*(7–15)*	*(8–16)*		
HD	30	30	15	14	1.00	14
	*(26–35)*	*(25–34)*	*(10–19)*	*(10–19)*		
FE	31	29	17	11	0.495	17
	*(28–37)*	*(23–32)*	*(20–11)*	*(8–17)*		
NA	27	25	13	8	0.604	9
	*(25–32)*	*(20–27)*	*(8–15)*	*(6–13)*		
SD	31	32	10	13	0.808	10
	*(26–34)*	*(29–37)*	*(17–15)*	*(8–16)*		
Performance-Enhancing Supplement						
OAE	22	21	14	18	0.641	14
	*(16–25)*	*(18–27)*	*(11–20)*	*(12–21)*		
HFI	30	15	9	10	0.171	7
	*(24–31)*	*(14–21)*	*(8–15)*	*(4–11)*		
**II**	**36**	**17**	**11**	**26**	**<0.001**	**17**
	***(23–32)***	***(21–30)***	***(15–24)***	***(13–22)***		
**HFE**	**23**	**21**	**7**	**27**	**0.005**	**13**
	***(13–21)***	***(23–31)***	***(9–17)***	***(17–25)***		
AG	23	22	16	21	0.512	17
	*(17–26)*	*(19–28)*	*(13–22)*	*(15–24)*		
HD	30	23	15	21	0.198	17
	*(26–35)*	*(25–34)*	*(10–19)*	*(10–19)*		
FE	24	25	24	15	0.285	17
	*(22–31)*	*(17–27)*	*(17–26)*	*(13–22)*		
NA	22	18	18	15	1.00	14
	*(18–26)*	*(14–22)*	*(14–22)*	*(11–19)*		
SD	26	28	15	17	1.00	15
	*(21–30)*	*(24–33)*	*(11–20)*	*(12–21)*		
Weight Loss Supplement						
OAE	3	9	33	30	0.117	5
	*(21–29)*	*(23–31)*	*(7–15)*	*(8–16)*		
HFI	4	4	35	21	0.701	3
	*(2–7)*	*(1–6)*	*(32–37)*	*(19–24)*		
II	10	3	37	40	0.073	6
	*(3–10)*	*(3–10)*	*(37–44)*	*(33–40)*		
HFE	4	12	26	36	0.260	6
	*(2–10)*	*(6–13)*	*(20–27)*	*(35–42)*		
AG	9	8	30	35	0.786	8
	*(4–12)*	*(5–13)*	*(27–35)*	*(30–38)*		
HD	11	6	34	38	0.281	8
	*(26–35)*	*(25–34)*	*(10–19)*	*(10–19)*		
**FE**	**15**	**2**	**33**	**38**	**0.002**	**7**
	***(22–31)***	***(18–27)***	***(17–26)***	***(13–22)***		
**NA**	**14**	**1**	**26**	**32**	**<0.001**	**6**
	***(5–12)***	***(3–10)***	***(28–35)***	***(23–30)***		
**SD**	**14**	**4**	**27**	**41**	**=0.007**	**8**
	*(5–12)*	*(6–13)*	*(29–36)*	*(32–39)*		

**Table 2:** Multivariable logistic regression models predicting supplement use. Odds ratios reflect unit increases in continuous predictors or category contrasts. Pseudo R^2^ values are reported as indices of model fit relative to null models and should not be interpreted as variance explained. Predictors were entered based on theoretical relevance.

**Model 1: Performance-Enhancing Supplements (PES)**						
**Predictor**	**Estimate (β)**	**SE**	**OR**	**95% CI** **(Lower)**	**95% CI** **(Upper)**	***p***
Intercept	−5.536	1.354	**—**	**—**	**—**	0.004
Investment in Ideals (II)	0.302	0.083	**1.34**	**1.14**	**1.58**	**<0.001**
Health-Fitness Evaluation (HFE)	0.215	0.074	**1.28**	**1.11**	**1.48**	**0** **.001**
Gender (male vs. others)	1.420	0.526	**3.40**	**1.53**	**7.54**	**0** **.003**
Model fit: Pseudo R^2^ = 0.19						
**Model 2: Weight-Loss Supplements (WLS)**						
**Predictor**	**Estimate (β)**	**SE**	**OR**	**95% CI** **(Lower)**	**95% CI** **(Upper)**	***p***
Intercept	−9.067	2.461	**—**	**—**	**—**	<0.001
Fatness Evaluation (FE)	0.309	0.080	**1.36**	**1.16**	**1.59**	**<0.001**
Age (years)	0.207	0.104	**1.23**	**1.00**	**1.51**	**0** **.046**
Model fit: Pseudo R^2^ = 0.22						

## Data Availability

The original contributions presented in the study are included in the article/Supplementary Materials, further inquiries can be directed to the corresponding author.

## Supplementary Materials

The following supporting information can be downloaded at: https://www.mdpi.com/article/10.3390/nu18010007/s1, File S1: Copy of survey completed by participants.

## Abbreviations

The following abbreviations are used in this manuscript:

DS	General Dietary Supplement
PES	Performance-Enhancing Supplement
WLS	Weight-Loss Supplement
BSIQ-SF	Body Self-Image Questionnaire-Short Form
OAE	Overall Appearance Evaluation (subscale)
HFI	Health Fitness Influence (subscale)
II	Investment in Ideals (subscale)
HFE	Health-Fitness Evaluation (subscale)
AG	Attention to Grooming (subscale)
HD	Height Dissatisfaction
FE	Fatness Evaluation
NA	Negative Affect
SD	Social Dependence
