# Supplementary material for: Exploration of Body Self-Image and Associated Body Composition Supplement Behaviors in College Students
Source: Nutrients. 2025 Dec 19;18(1):7. doi: 10.3390/nu18010007 (PMC12787663; doi:10.3390/nu18010007)
Supplement: Supplementary file 1 [file nutrients-18-00007-s001.zip › nutrients-4026727-supplementary.pdf]

Supplementary Materials.

File S1. Copy of survey completed by participants

Body Self-Image Questionnaire – Short Form (BSIQ-SF) as previously published: Rowe, D. Factorial validity and cross-validation of the Body Self-Image Questionnaire (Short Form) in young adults. In: American College of Sports Medicine National Convention, Nashville, TN, USA (June 2005).

BSIQ-SF Responses were presented as:

a) Not at all true of myself
b) Slightly true of myself
c) About halfway true of myself
d) Mostly true of myself
e) Completely true of myself

BSIQ-SF Questions

1) I think my body is unattractive.
2) How well my body is functioning influences the way I feel about my body.
3) Having a well-proportioned body is important to me.
4) My overall fitness level is high.
5) I compare my body to people I'm close to (friends, relatives, etc)
6) I've often wanted to be taller.
7) I think my body looks fat in clothes.
8) My naked body makes me feel sad.
9) I pay careful attention to my face and hair, so that I will look good.
10) I look good in clothes.
11) I feel better about my body when I'm fitter.
12) Body size matters to me.
13) My body is healthy.
14) Being around good-looking people makes me feel bad about my body.
15) I wish I were a different height.
16) My body is overweight.
17) I feel depressed about my body.
18) I'm usually well-dressed.
19) My body looks good.
20) The way I feel about my body improves when I exercise regularly.
21) I care about how well-shaped my legs are.
22) My body is in shape.
23) I'm more aware of my body when I'm in social situations.

Supplementary Materials.

24) If I were a different height, I'd like my body better.
25) I wish I were thinner.
26) Most days I feel bad about my body.
27) I spend time making my appearance more attractive.

Supplement use responses were presented as:

a) YES
b) NO

Supplement questions

1) Are you currently taking, or in the past year taken a Vitamin or Mineral supplement? *Examples: Multivitamin, or individual vitamins like B-Complex, B12, B6, C, A, D, E, K. Minerals, like Calcium, Magnesium, Potassium, Iron or Zinc*.
2) Are you currently taking, or in the past year have taken a Performance-Enhancing Supplement?
Example: Creatine, Beta-Alanine, BCAA'S (Branched-Chain Amino Acids) or Caffeine pills.
3) Are you currently taking, or in the past year have taken, a Weight Loss Supplement? *Example: Appetite suppressant, Thermogenic agents like caffeine pills or green tea extract or Fat blockers*.
